# Large outbreak of typhoid fever on a river cruise ship used as accommodation for asylum seekers, the Netherlands, 2022

**DOI:** 10.2807/1560-7917.ES.2024.29.5.2300211

**Published:** 2024-02-01

**Authors:** Daisy Ooms, Anne de Vries, Femke DH Koedijk, Ellen Generaal, Ingrid HM Friesema, Maxine Rouvroye, Steven FL van Lelyveld, Maaike JC van den Beld, Daan W Notermans, Patrick van Schelven, Janine FH van den Brink, Tanja Hartog, Thijs Veenstra, Serena Slavenburg, Jan C Sinnige, Wilhelmina LM Ruijs

**Affiliations:** 1 Centre for Infectious Disease Control, National Institute for Public Health and the Environment (RIVM), Bilthoven, the Netherlands; 2 Department of Communicable Disease Control, Public Health Service of Kennemerland, Haarlem, the Netherlands; 3 Department of Communicable Disease Control, Public Health Service of Twente, Enschede, the Netherlands; 4 Department of Infectious Diseases, Public Health Service of Amsterdam, Amsterdam, the Netherlands; 5 Department of Internal Medicine, Spaarne Gasthuis, Haarlem/Hoofddorp, the Netherlands; 6 Department of Communicable Disease Control, Public Health Service of Gelderland-Midden, Arnhem, the Netherlands; 7 Department of Communicable Disease Control, Public Health Service of IJsselland, Zwolle, the Netherlands; 8 Regional Public Health Laboratory Kennemerland, Haarlem, the Netherlands

**Keywords:** *Salmonella* Typhi, typhoid fever, outbreak, asylum seeker, refugee, migrant, shelter, river cruise ship, wastewater leak, water quality, the Netherlands

## Abstract

On 6 April 2022, the Public Health Service of Kennemerland, the Netherlands, was notified about an outbreak of fever and abdominal complaints on a retired river cruise ship, used as shelter for asylum seekers. The diagnosis typhoid fever was confirmed on 7 April. An extensive outbreak investigation was performed. Within 47 days, 72 typhoid fever cases were identified among asylum seekers (n = 52) and staff (n = 20), of which 25 were hospitalised. All recovered after treatment. Consumption of food and tap water on the ship was associated with developing typhoid fever. The freshwater and wastewater tanks shared a common wall with severe corrosion and perforations, enabling wastewater to leak into the freshwater tank at high filling levels. *Salmonella* Typhi was cultured from the wastewater tank, matching the patient isolates. In the freshwater tank, *Salmonella* species DNA was detected by PCR, suggesting the presence of the bacterium and supporting the conclusion of contaminated freshwater as the probable source of the outbreak. Outbreaks of uncommon infections may occur if persons from endemic countries are accommodated in crowded conditions. Especially when accommodating migrants on ships, strict supervision on water quality and technical installations are indispensable to guarantee the health and safety of the residents.

**Key public health message**

**What did you want to address in this study and why?**

Typhoid fever is a severe disease caused by the bacterium *Salmonella* Typhi. It spreads via faecal contamination of food or water and is common in countries with poor sanitation. In April 2022, a large outbreak of typhoid fever among asylum seekers housed on a retired ship was reported in the Netherlands. We investigated the outbreak, performed a survey among people living and working on the ship, inspected the ship and took water samples from various places on the ship and analysed them.

**What have we learnt from this study?**

The cause of this outbreak was a technical defect in the ship’s construction. The wastewater tank and the freshwater tank shared a common wall, which was perforated due to severe corrosion. This enabled sewage water contaminated with *Salmonella* Typhi, leaking into the freshwater tank, consequently infecting people who consumed this water.

**What are the implications of your findings for public health?**

Strict supervision of water quality and technical installations is key to the health and safety of the persons on ships, especially in crowded living conditions. Water tanks and associated equipment must be properly maintained and regularly inspected, especially on older ships.

## Background

Typhoid fever is a severe systemic disease caused by *Salmonella enterica* subspecies *enterica*, serovar Typhi (*S*. Typhi) [1,2]. After an incubation period of 7 to 14 days (range: 3–60 days), the disease is characterised by a bacteraemia with high fever up to 3 weeks [1,2]. Fifty percent of the patients have gastrointestinal symptoms [3]. Severe complications occur in 10–15% of the cases, varying from ileal perforation, encephalopathy, meningitis, myocarditis, osteomyelitis, hepatitis, shock and miscarriage [2,4-8]. Adequate antimicrobial treatment reduces mortality rates from 15 to 1% [2]. One to 4 percent of infected persons develop an asymptomatic chronic carrier status, defined as shedding *S*. Typhi 12 months after the initial infection. Gallstones serve as a reservoir and are found in 90% of chronic carriers [9].

Diagnosis of typhoid fever requires detection of *S*. Typhi, usually from blood or stool. Culture of *S.* Typhi from blood or stool is not highly sensitive. Due to irregular shedding of bacteria in stools, faecal cultures are positive in only 30–40% of patients with typhoid fever, requiring repeated testing to detect carriers [2,10,11].

Humans are the only reservoir of *S*. Typhi with infections mainly acquired through ingestion of contaminated water or food [1]. The bacterium may survive days to weeks in water [12,13] and is endemic in countries with unsafe water, inadequate sanitation and poor hygiene, with highest transmission in densely populated areas and crowded households [14-16]. Direct human-to-human transmission is uncommon but has been reported [17]. The World Health Organization (WHO) estimates the annual number of cases to 11–20 million, resulting in ca 128,000–161,000 deaths yearly [18,19]. Highest incidences are in the Indian subcontinent, Southeast Asia and sub-Saharan Africa [19].

In non-endemic countries, typhoid fever cases are few and mainly sporadic [19] and the impact on public health is limited. The annual incidence in the Netherlands amounted 0.11 cases per 100,000 inhabitants over the last decade [20,21], in the European Union/European Economic Area (EU/EEU) countries 0.19 cases per 100,000, with 90.9% of cases being travel-related [22]. Secondary transmission rates are low, even among household contacts [23].

In this report we describe a large outbreak on a retired river cruise ship used as accommodation for asylum seekers. Two ships were involved, each sheltering ca 100 asylum seekers, sharing one galley for preparation of food.

## Outbreak detection

On 6 April 2022, the hygiene department of the Public Health Service (PHS) of Kennemerland was contacted by the Central Agency for the reception of Asylum seekers (COA) and the Asylum seeker Healthcare Organisation (GZA). Concerns about the water quality on a ship, used as accommodation for asylum seekers, were raised in combination with reported illness in several individuals. Stool samples of several patients had been collected for diagnosis of enteric pathogens, but results were not yet reported. An extensive outbreak investigation was performed, including an epidemiological survey and technical and microbiological investigations of the ship.

## Methods

### Setting

After the first notification, the ship hygiene inspector of the PHS visited the location. The ship (Ship 1) was a former river cruise ship, built in 1939, used to accommodate ca 100 male asylum seekers. In addition, another 100 male asylum seekers from an adjacent ship (Ship 2) had eaten their meals on Ship 1 since 30 March.

**Figure 1:**
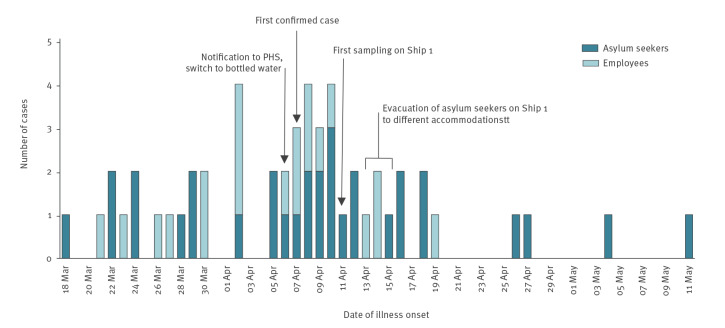
Epidemic curve of symptomatic, confirmed cases of *Salmonella* Typhi in an outbreak of typhoid fever on two ships used as accommodation for asylum seekers, the Netherlands, March–May 2022 (n = 52)

Since 24 March, 23 individuals had reported complaints of high fever lasting up to 2 weeks, malaise, headache, nausea, vomiting, abdominal pains and diarrhoea. Most cases (15/23) were reported in the week before 7 April (Figure 1). The diagnosis typhoid fever was first confirmed by blood culture in a hospitalised patient on 7 April and reported to the PHS as a notifiable disease, in accordance with Dutch legislation.

PHS: Public Health Service.

A total of 52 of the 72 identified cases had clinical symptoms. Only symptomatic cases were included in this epidemiological curve.

### Outbreak case definition

Cases of typhoid fever were categorised as possible, suspected or confirmed (Box). Initially, the time period was set to 1 March–15 April but it was extended backwards to 12 February when previously unrecognised patients, with an earlier onset of disease, were identified during the outbreak investigation.

Box **Definitions of possible, suspected and confirmed cases of *Salmonella* Typhi infection, outbreak of typhoid fever on two ships used as accommodation for asylum seekers, the Netherlands, March–May 2022**

**Possible case:**

A person who consumed meals or tap water on Ship 1 between 12 February and 15 April 2022, either not tested or *Salmonella* not detected by PCR or culture.

**Suspected case:**

A person who consumed meals or tap water on Ship 1 between 12 February and 15 April 2022 in combination with *Salmonella* species detected from a stool sample by PCR, but *Salmonella* Typhi not detected by culture.

**Confirmed case:**

A person who consumed meals or tap water on Ship 1 between 12 February and 15 April 2022, with *Salmonella* Typhi detected by culture from a blood or stool sample.

### National case register and epidemiological data

Since typhoid fever is a notifiable disease, the PHS performs source and contact tracing and gathers information on basic demographics and disease outcome. For national surveillance purposes, the PHS reports anonymised data to the National Institute for Public Health and the Environment (RIVM) of the Netherlands, including demographic variables and disease specific information.

### Epidemiological survey

#### Questionnaire for source tracing

An anonymous survey among asylum seekers and employees (e.g. maritime personnel living on the ship and dayworkers such as cleaners, repair workers, security guards) in the accommodation linked to the outbreak was conducted, either on-site or by phone. The inclusion criterium was presence on either one of the ships between 1 March and 15 April 2022. Questions included sociodemographic variables, ship of residence, symptoms and test results for *S*. Typhi and the time period and frequency of food and tap water consumption on Ship 1. Consumption of food and tap water was assessed as a dichotomous variable (yes/no). To assess the frequency of food consumption, meals (breakfast, lunch, dinner) were inventoried separately. The questionnaire was drafted in Dutch and English. Translators were available for translation to the native language (Arabic, Dari, Farsi, French, German, Kurdish, Mandinka, Punjabi, Somali, Tigrinya, Turkish) of the asylum seekers.

#### Data analyses

Descriptive statistics were reported as median or percentage. An epidemiological curve was plotted to show the distribution of cases over time, based on the date of onset of disease (Figure 1). Consumption of food and tap water on the ship was compared between cases and non-cases, separately for asylum seekers and employees. Odds ratios (OR) and 95% confidence intervals (CI) were calculated using univariable regression to test for differences in proportions, using IBM SPSS Statistics, version 28 (IBM New York, United States (US)).

### Technical inspection of Ship 1

In April and May, six investigation visits to Ship 1 took place, performed by ship technicians, public health and laboratory specialists, accompanied by ship staff (Figure 1). The galley was inspected, and food safety and cleaning procedures were evaluated. No technical information regarding the freshwater system and piping of the ship was available. Therefore, the location of freshwater and wastewater system, corresponding inlets and outlets and all tap points were mapped. Freshwater tanks were entered to inspect the condition of the tank walls. Only limited inspection was possible, as a full search would have required dismantling of the ship.

### Microbiological analyses

#### Specimens from humans

All asylum seekers and food handlers working on Ship 1, who met the case definition for a possible case were requested to leave a stool sample. Persons with symptoms of fever were referred for blood culture. First, PCR screening on *Salmonella* species was performed. If positive, followed by a culture on *S.* Typhi. If negative, a second stool sample was requested after 4 weeks.

Blood and stool samples from patients with suspected typhoid fever were examined for *S.* Typhi in 15 different microbiology laboratories. Blood cultures were collected at Streeklab Haarlem, the Netherlands in Bactec bottles and incubated in a Bactec FX (Becton Dickinson, Franklin Lakes, US) at 35°C for 5 days.

Faecal samples were analysed for *Salmonella*, *Shigella* and *Campylobacter* species using a multiplex quantitative PCR (qPCR), focusing on the *ttr* gene as a pan-*Salmonella* target (QIAsymphony, LightCycler 480, Qiagen, Hilden, Germany) [24]. Stool samples positive for PCR were cultured on selective agars, Brilliant Green Agar (BGA), Salmonella-Shigella agar (SS), Sorbitol McConkey agar (MAC-S) and a selective enriched Selenite Cysteine broth (SEL), incubated at 35°C overnight and then observed for growth at 24 and 48 hours. All selective media were from Thermo Fisher Scientific, Waltham, US.

Identification of *Salmonella* species was confirmed by MALDI-TOF MS (Bruker, Billerica, US). Serotyping was performed using an agglutination test according to Remel^tm^ Agglutinating Sera (Thermo Fisher Scientific, Waltham, US). Minimum inhibitory concentrations (MIC) were determined by automatic susceptibility systems (Vitex 2XL, BioMérieux, Marcy-l'Étoile, France). All isolates were sent to RIVM for confirmation and whole genome sequencing.

#### Environmental samples

During the technical inspections, 40 environmental samples were taken from freshwater taps of the bar, cabins, public toilets and standing water in drain wells and kitchenware in the galley. Furthermore, samples were taken from freshwater and wastewater tanks and corresponding in- and outlets. In Supplement 1, a detailed overview of the exact locations of the sampling points are indicated on the horizontal cross section of Ship 1.

All environmental samples were enriched in Selenite Cystine broth (SEL) (Thermo Fisher Scientific), incubated at 35°C for 24 hours and 30 µL of the incubated broth was spread onto BGA, SS and MAC-S and incubated at 35°C for 48 hours. A multiplex *Salmonella* qPCR was performed directly on the enriched broth and on suspected colonies on selective agars. Water samples were collected (0.5–5 L) from four freshwater and wastewater tanks and the six samples (500 mL) were filtered through a polycarbonate membrane filter with pore size 0.2 µm (Whatman, Cytiva, US) to collect and concentrate bacterial cells. Each filter was transferred to petri dishes containing 1 mL phosphate buffered saline (PBS), after which 0.5 mL of the suspension was transferred into 10 mL SEL and further analysed as described above. The membrane filter was cultured on R2A agar (Thermo Fisher Scientific). Simultaneously, a multiplex *Salmonella* qPCR was performed on the concentrate. Isolates suspected of *Salmonella* species were identified by MALDI-TOF and agglutination tests. Isolates of *S.* Typhi were sent to the RIVM for genotyping.

### Genotyping

All isolates were whole genome sequenced (WGS) using Illumina technology (Illumina Inc, San Diego, US) to generate paired end reads. Accredited sequencing and processing raw reads into de novo assemblies, in silico *Salmonella* serotyping and cluster-analysis using core genome MLST was performed [25]. Antimicrobial resistance markers were detected with an in-house developed pipeline using ResFinder (https://cge.food.dtu.dk/services/ResFinder/) and PointFinder (https://bio.tools/PointFinder) databases. One of the representative genomes of the *S.* Typhi cluster was further typed using GenoTyphi [26,27]. Global relatedness with other *S.* Typhi isolates was assessed by importing a representative genome in the EnteroBase database [28,29]. Raw sequence data were submitted to the European Nucleotide Archive (ENA) (https://www.ebi.ac.uk/ena/browser/home) under study number PRJEB54812.

## Results

### Description of cases

A total of 365 persons (228 asylum seekers, 137 employees) had consumed meals or tap water on Ship 1, between 12 February and 15 April 2022. Of these, 72 (20%) persons (52 asylum seekers, 20 employees) met the case definition for a confirmed case and five (1%) (four asylum seekers, one employee) were classified as suspected cases. The other 288 (79%) (172 asylum seekers, 116 employees) were exposed, but 132 of them tested PCR negative for *Salmonella* species (spp.) and 156 were not tested (Table 1). No secondary cases were detected.

**Table 1:** Confirmed, suspected and possible cases of *Salmonella* Typhi infection in an outbreak of typhoid fever on a ship used as shelter for asylum seekers, the Netherlands, March–May 2022 (n = 365)

Cases	Asylum seekers (n = 228)		Employees (n = 137)	
	n	%	n	%
Confirmed case	52	23	20	15
Suspected case	4	2	1	1
Possible case tested, *Salmonella* not detected	113	50	19	14
Possible case, not tested^a^	59	26	97	71

^a^ Not tested: test not indicated, not willing to test or lost to follow-up.

Most asylum seekers came from countries in southwest Asia (28/52), followed by Africa (12/52) (Table 2). Most of the infected employees were Dutch (11/20) or came from other European countries (6/20). Most frequently reported symptoms were fever (60%), gastro-intestinal complaints (58%) and headache (56%).

**Table 2:** Sociodemographic characteristics of asylum seekers (n = 52) and employees (n = 20) testing positive for *Salmonella* Typhi^a^ in an outbreak of typhoid fever on a ship used as shelter for asylum seekers, the Netherlands, March–May 2022 (n = 72)

Characteristics	Asylum seekers	Employees
Sex		
Male	52	17
Female	0	3
Age (in years)		
Median (IQR)	30 (25.0–36.3)	31 (28.0–41.5)
Country or region of birth		
The Netherlands	0	11
Southwest Asia^b^	28	0
Africa	12	3
Other European countries	1	6
Unknown	11	0
Self-reported symptoms related to *Salmonella* Typhi		
Diarrhoea/constipation/malaise/stomach pain	24	18
Fever	27	16
Headache	24	16
Non-productive cough	7	4
No symptoms	11	0
Unknown	9	0
Hospital admissions due to *Salmonella* Typhi		
Yes	13	12
No	30	8
Unknown	9	0

IQR: interquartile range.

^a^ *Salmonella* Typhi detected from blood or faecal specimen.

^b^ Southwest Asia: Saudi Arabia, Syria, Turkey, Yemen and Pakistan.

Source: OSIRIS (national registration system for notifiable infectious diseases in the Netherlands).

A total of 13 asylum seekers and 12 employees were hospitalised in different hospitals in the Netherlands. All patients recovered. Most were treated with oral ciprofloxacin for 14 days. Some hospital-admitted patients received ceftriaxone intravenously, followed by oral therapy with ciprofloxacin or trimethoprim-sulfamethoxazole. One patient was re-admitted after 5 days with cholecystitis for which a cholecystectomy was performed.

### Epidemiological survey

In total, 155 (68%) asylum seekers filled in the questionnaire (Table 3). There was a significant difference in attack rate between asylum seekers accommodated exclusively on Ship 1 (25/72 tested positive; attack rate 35%) and those housed exclusively on Ship 2 (6/73; attack rate 8%) (p < 0.001). Ten asylum seekers indicated to have lived on both ships. The overall attack rate was 23% (36/155). Cases reported consumption of lunch (OR = 4.2; 95% confidence interval (CI): 1.6–10.6) and tap water (OR = 2.4; 95% CI: 1.0–5.8) more frequently than non-cases.

**Table 3:** Consumption of food and water by asylum seekers during an outbreak of typhoid fever on two ships used as accommodation for asylum seekers the Netherlands, March–May 2022 (n = 155)

Exposures	*S*. Typhi detected^a^ (n = 36)^b^	*S*. Typhi not detected (n = 119)^b^	OR	95%CI
	n	n		
Consumption of food				
Yes	33	92	3.2	0.9–11.4
No	3	27		
Frequency breakfast				
Daily or almost daily	19	54	0.9	0.4–2.1
Sometimes or never	14	37		
Frequency lunch				
Daily or almost daily	25	42	**4.2**	**1.6–10.6**
Sometimes or never	7	49		
Frequency dinner				
Daily or almost daily	29	69	3.2	0.9–11.6
Sometimes or never	3	23		
Tap water				
Yes	26	68	**2.4**	**1.04–5.8**
No	8	51		
Frequency tap water				
More than weekly	25	65	1.2	0.1–11.2
Weekly or less	1	3		

OR: odds ratio; *S*.: *Salmonella.*

^a^ *Salmonella* Typhi detected from a blood or faecal specimen.

^b^ Total number of responses may vary due to missing values.

Entries in bold represent statistically significant findings.

The questionnaire was filled in by 79 employees (58%) (Table 4). Half of the employees worked on both ships and no significant difference was found in attack rates between employees who worked exclusively on either one of the ships. On Ship 1, cases reported consumption of breakfast (OR = 7.1; 95% CI: 1.4–35.6) and lunch (OR = 5.1; 95% CI: 1.01–25.6) more frequently than non-cases (p = 0.0001 and p = 0.05, respectively). Since all cases had dinner on the ship, this was not tested. No difference between cases and non-cases was found in consumption of tap water.

**Table 4:** Consumption of food and water during an outbreak of typhoid fever by employees on two ships used as accommodation for asylum seekers, the Netherlands, March–May 2022 (n = 79)

Exposures	*S*. Typhi detected^a^ (n = 21)^b^	*S*. Typhi not detected (n = 58)^b^	OR	95%CI
	n	n		
Consumption of food				
Yes	19	39	4.6	0.98–22.0
No	2	19		
Frequency breakfast				
Daily or almost daily	15	19	**7.1**	**1.4–35.6**
Sometimes or never	2	18		
Frequency lunch				
Daily or almost daily	16	22	**5.1**	**1.01–25.6**
Sometimes or never	2	14		
Frequency dinner				
Daily or almost daily	18	22	NA	NA
Sometimes or never	0	16		
Tap water				
Yes	5	19	0.9	0.3–2.8
No	12	39		
Frequency tap water				
More than weekly	4	14	2.0	0.2–20.9
Weekly or less	1	5		

NA: not applicable; OR: odds ratio.

^a^ *Salmonella* Typhi detected from a blood or faecal specimen. One employee without a positive *S*. Typhi culture self-reported as positive. Since the questionnaire was anonymous, it was not possible to correct afterwards.

^b^ Total number of responses may vary due to missing values.

Entries in bold represent statistically significant findings.

### Technical inspection of Ship 1

Ship 1 was built in 1939 as a cargo ship and modified in 1973 to accommodate passengers and showed signs of intensive use. Meals were prepared in the galley, using freshwater from taps. Hygiene and cleaning protocols were available in written procedures and orally confirmed by galley staff.

No information was available on the current technical layout of Ship 1, neither a map indicating the position of piping and tanks. Only one freshwater tank manhole was directly visible, leading to a starboard tank positioned under the dining area.

An old indicating panel suggested the presence of more tanks. After removing carpets and furniture, three additional manholes were found, compatible with four freshwater tanks. One large wastewater tank was found, bordering the freshwater tanks and located towards the stern. The shape of the wastewater tank suggested that it had been enlarged towards the bow of the ship, now occupying a crawl space between the freshwater tanks, directly against the freshwater tanks and sharing a common wall. The wastewater tank was covered with a zinc plate to cover holes in the original roof.

Inspection of the freshwater tanks revealed severe corrosion in the walls. Small perforations were found in the wall, towards the top of the freshwater tanks that bordered the wastewater tank. One of these perforations seemed wet, although located above the water level at the time of inspection. The shared wall between the freshwater and wastewater tank was permeable, enabling wastewater to leak into the freshwater tank, causing contamination of freshwater with wastewater. In Supplement 1, a horizontal and vertical cross-section of Ship 1 shows how the freshwater and wastewater tanks were situated, sharing a common wall. The pictures in Supplement 2 show evidence of perforations in the walls of the tanks.

### Microbiological analyses

#### Specimens from humans

A total of 77 patients tested PCR positive for *Salmonella* spp., of which 72 were confirmed by a culture of *S*. Typhi (Table 1). Three cases were identified during the second PCR screening, four weeks after these patients tested negative in the initial PCR testing.

In total, 156 persons (59 asylum seekers, 97 employees) were not tested for *Salmonella* spp. with PCR, either because they were not willing to participate or because they were lost to follow-up (Table 1). The latter was mainly the case for employees, whose contact details were not available.

#### Environmental samples

Forty samples taken from freshwater taps, drain wells, cabins and public toilets all contained environmental bacteria. Some galley ware and drain well samples contained Enterobacterales, however, *Salmonella* species were not detected with PCR. One sample taken from the wastewater tank was *Salmonella* spp. PCR positive and *S*. Typhi was cultured from filtered wastewater.

Enterobacterales were detected from two samples from freshwater tanks, which is an indicator for faecal contamination. Four of the freshwater samples were *Salmonella* spp. PCR positive but the presence of *S*. Typhi could not be confirmed by culture. Sequencing of the samples was not considered possible, due to low numbers of DNA copies.

### Genotyping

In total, RIVM received 84 isolates from 72 patients from 15 clinical microbiological laboratories. Additionally, one isolate cultured from the wastewater tank was received.

**Figure 2:**
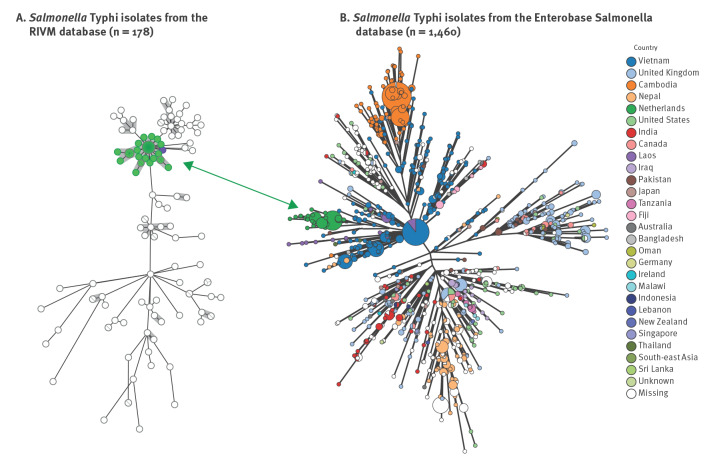
Minimum spanning tree of *Salmonella* Typhi isolates in RIVM since 2021 and EnteroBase databases, accessed 8 September 2023 (n = 1,460)

RIVM: National Institute for Public Health and the Environment; ST: sequence type.

The serovar Typhi was confirmed, and the isolates were sequence type (ST) 1 (cgMLST). All isolates possessed the *aac(6’)-Iaa* gene, associated with aminoglycoside resistance, which is a cryptic chromosomal-encoded aminoglycoside acetyltransferase gene found in all *Salmonella* serovars. No further resistance markers were detected. Using cgMLST, all isolates formed one large cluster within five alleles distance. The cluster did not relate to other *S.* Typhi isolates present in the RIVM database with 178 *S*. Typhi isolates since 2021 (Figure 2A). Hierarchical clustering in EnteroBase indicated that the isolates belonged to cluster 6578 on level HC5. In this HC5:6578 are 1,460 genomes from the global *Salmonella* database present, including those from the 85 isolates from this survey (accessed 8 September 2023, Figure 2B). According to GenoTyphi, the isolates belonged to subclade 4.3.1.1, which is mostly found in South Asia, Southeast Asia and East Africa [26].

EnteroBase is a public database that supports the identification of global population structures within several bacterial genera.

A. A tree from 178 *Salmonella* Typhi, ST1 and ST2 isolates from the Dutch RIVM database. Green nodes indicate human isolates epidemiologically linked to the outbreak. The dark purple node indicates the isolate from the wastewater tank. White nodes represent other isolates in the RIVM database. A grey halo indicates a cluster with cut-off five alleles.

B. A tree from 1,460 global isolates with HC5:6578 from EnteroBase *Salmonella* database. Green colour indicates the 85 Dutch genomes from this outbreak.

## Outbreak control measures

Immediately after the first notification of concerns about the water quality on 6 April, preventive measures were taken. The hygiene inspector of PHS recommended use of bottled water until water quality was tested and proven to be safe. Advice about personal hygiene, food preparation and cleaning procedures was given.

On 15 April, all asylum seekers were evacuated from the two ships. Persons with clinical symptoms, with a positive test result or who were roommates of confirmed cases were placed in an isolation location with private sanitary facilities. Persons without a positive test and without symptoms were housed in three other accommodations, spread across the Netherlands.

Coordination was required to manage this outbreak on a national level. An outbreak response team was established to align outbreak control measures and diagnostic and therapeutic policies. Collaboration between relevant stakeholders, such as PHSs, COA, GZA, general practitioners, infectious disease specialists, hospitals and clinical microbiologists, was established.

Before arriving at the alternative shelter locations, the PHS informed general practitioners and the GZA about the outbreak to raise awareness of relevant symptoms and align diagnostic policies. Shared bathrooms were locked, and food was catered externally.

The PHS provided health education to asylum seekers and staff in small groups with a translator. This included disease specific information, transmission routes and relevant hygienic prevention measures. Furthermore, the PHS provided stool sample containers for all asylum seekers, to screen for *Salmonella* spp. and if positive, additional culture for *S*. Typhi was performed. Severely ill patients were referred to the hospital.

Outbreak investigation was challenging since many relocations of asylum seekers took place in the period before and during the outbreak. Additionally, physical presence at the accommodation was not registered. Asylum seekers who resided elsewhere could not be contacted, since address and contact details were unknown. A notable part of the maritime personnel, mostly contract workers from countries in eastern Europe, were contracted via employment companies. Persons, whose contact details were available, were informed about exposure risks and advised to be tested.

As potentially exposed employees might return to their home countries, an alert was sent via the Early Warning and Response System (EWRS) of the European Union and a request to pass on information about possible cases on 2 May 2022. No further cases were reported from abroad.

Because of the risk of further transmission in crowded shelters, all asylum seekers with proven and suspected *S*. Typhi infection were advised screening for bacteriological clearance by three consecutive stool samples (24 hours apart) and at least 2 weeks after completion of antimicrobial therapy. Exposed asymptomatic individuals were advised screening at the end of the incubation period.

In case of detection of *S*. Typhi in stools, treatment with ciprofloxacin (500 mg twice a day for 14 days) or azithromycin (1,000 mg/d for 7 days) was advised, in accordance with national guidelines for typhoid fever.

## Discussion

We describe an outbreak of *S.* Typhi among asylum seekers and employees on a retired river cruise ship, used as shelter for asylum seekers, in the Netherlands.

To determine the cause of the outbreak an epidemiological survey was performed, as well as technical inspection and environmental sampling of the ship and sequencing of *S*. Typhi. We conclude that the most probable source of this outbreak was leakage of contaminated wastewater from the wastewater tank into the freshwater tank resulting in infections of asylum seekers and employees and consequently enhanced excretion of *S*. Typhi in wastewater.

The results of the epidemiological survey indicated that frequent consumption of food (asylum seekers and employees) and tap water (asylum seekers) on board significantly related to the risk of developing typhoid fever. Tap water was used for the preparation of meals. An in-depth investigation on consumption of specific food items was not considered reliable because of recall bias. Since asylum seekers and staff were not obliged to stay in the shelters and physical presence was not registered, the duration of stay could not be determined with certainty. Therefore, it was not possible to investigate the length of stay on the ships in relation to the risk of illness. Other limitations were that not all asylum seekers and staff completed the questionnaire, some staff members could not be reached and the reasons for non-participation are unknown. The findings on food and water consumption should therefore be interpreted with caution.

The cgMLST clustering proved that patients belonged to the same outbreak. Subclade 4.3.1.1. is a genotype that is frequently isolated from several countries in South Asia, Southeast Asia and East Africa. The asylum seekers were of 20 different nationalities, including countries in which subclade 4.3.1.1. is endemic. Therefore, in this outbreak, the genotype is not informative for tracing the geographical source.

Investigation on ships requires specific knowledge of technical constructions, especially in case of technically outdated, adjusted ships, without technical blueprints. Mapping of water tanks, piping, and inlets and outlets was very complex and time-consuming. Consequently, sampling of water tanks was performed when asylum seekers were already relocated and therefore further addition of *S*. Typhi in the wastewater by infected persons had ceased. Furthermore, the freshwater had been chlorinated before sampling was performed. It is difficult to detect *S*. Typhi from water, even in endemic locations [12,30] and the presence of *S*. Typhi in the freshwater tank could not been confirmed. However, other observations strongly support the assumption that leakage from wastewater with *S*. Typhi into the freshwater tanks, must have been the most likely route of transmission: (i) samples from the wastewater tank were positive for *S*. Typhi, matching the outbreak strain in patients, (ii) *Salmonella* spp. were found in all four freshwater tanks, together with other Enterobacterales, which indicate faecal contamination, (iii) visible evidence of holes, caused by severe corrosion, in the common wall between the wastewater and freshwater tanks, enabling water to leak from the wastewater tank into the freshwater tank. The epidemiological curve suggested prolonged transmission that could be explained by intermittent leakage because leakage was only possible at high filling levels of the wastewater tank. As the wastewater tank was frequently drained when full, leakage then temporarily stopped until high filling levels were reached again.

The fact that the outbreak occurred among asylum seekers accommodated and contract workers of different nationalities and several relocations, complicated outbreak control.

Although the capacity of the ship was ca 100 persons, the residents of an adjacent ship also ate their meals on the first ship, starting from 30 March, bringing the total number of asylum seekers at risk to 228 persons. In addition, an unknown number of employees lived on either of the ships during the time period and several repair workers also received meals from the ships’ galley. The number of different companies and international employment agencies, and absence of contact details made tracing of exposed persons difficult.

In the Netherlands, several organisations are involved in the healthcare for asylum seekers. At the beginning of this outbreak, communications between stakeholders were challenging; the relocation of asylum seekers was decided upon very short notice with insufficient time to properly prepare infection prevention measures at shelter locations. Different priorities between public health authorities and the authorities responsible for the accommodation of asylum seekers, complicated effective outbreak control.

In the European Union, the number of first-time asylum applicants in 2021 amounted 535,000, with Syrians (18.4%), Afghans (15.6%), Iraqis (4.9%) and Pakistani (3.9%) as the most common nationalities [31]. In all these countries typhoid fever is endemic [19]. However, mandatory stool screening in newly arrived asylum seekers showed low prevalence of enteric bacteria, such as *Salmonella* spp [32,33]. Occasional introduction of *S*. Typhi is nevertheless possible. However, reported outbreaks of *S*. Typhi in Europe are rare [15-17,19] and secondary transmission rates are low, even among household contacts [23], sheltering large numbers of refugees in densely populated locations with shared sanitary and kitchen facilities may lead to outbreaks.

## Conclusion

In the Netherlands, ships are increasingly used to accommodate asylum seekers. This outbreak indicates that current regulations may not be adequate to guarantee the health and safety of residents. In our case, the ship possessed the mandatory technical certificate (CvO) for river cruise ships, issued every 5 years. However, according to regulations from 1996, freshwater tanks on ships are not allowed to share a single wall with wastewater tanks. A transitional arrangement of 10 years was agreed for existing ships. The ship in question appears to have received an unjustified certification and because of the lack of technical blueprints no correct assessment was possible and no dedicated modifications could be required. In response to this outbreak, the Ministry of Infrastructure and Water Management and The Human Environment and Transport Inspectorate, which supervises the certification agencies, were informed to create awareness on possible similar technical deficiencies in other ships. In case of use of retired (river) cruise ships as temporary shelters, technical inspection is essential to prevent waterborne diseases. Especially when asylum seekers are accommodated in these crowded conditions, uncommon pathogens may be introduced in these communities resulting in outbreaks.

## Supplementary Data

603000 Supplementary Material
