# Supplementary material for: Large outbreak of typhoid fever on a river cruise ship used as accommodation for asylum seekers, the Netherlands, 2022
Source: Euro Surveill. 2024 Feb 1;29(5):2300211. doi: 10.2807/1560-7917.ES.2024.29.5.2300211 (PMC10835751; doi:10.2807/1560-7917.ES.2024.29.5.2300211)

Supplementary material

This supplementary material is hosted by Eurosurveillance as supporting information alongside the article '*Large outbreak of typhoid fever on a river cruise ship used as accommodation for asylum seekers, the Netherlands, 2022'*, on behalf of the authors, who remain responsible for the accuracy and appropriateness of the content. The same standards for ethics, copyright, attributions and permissions as for the article apply. Supplements are not edited by Eurosurveillance and the journal is not responsible for the maintenance of any links or email addresses provided therein.

Appendix 1: blueprints of the ship

Map 1: horizontal cross-section of the ship

Blueprint of the first deck of the ship, with brown colour indicating the wastewater tank and in green the freshwater tanks.

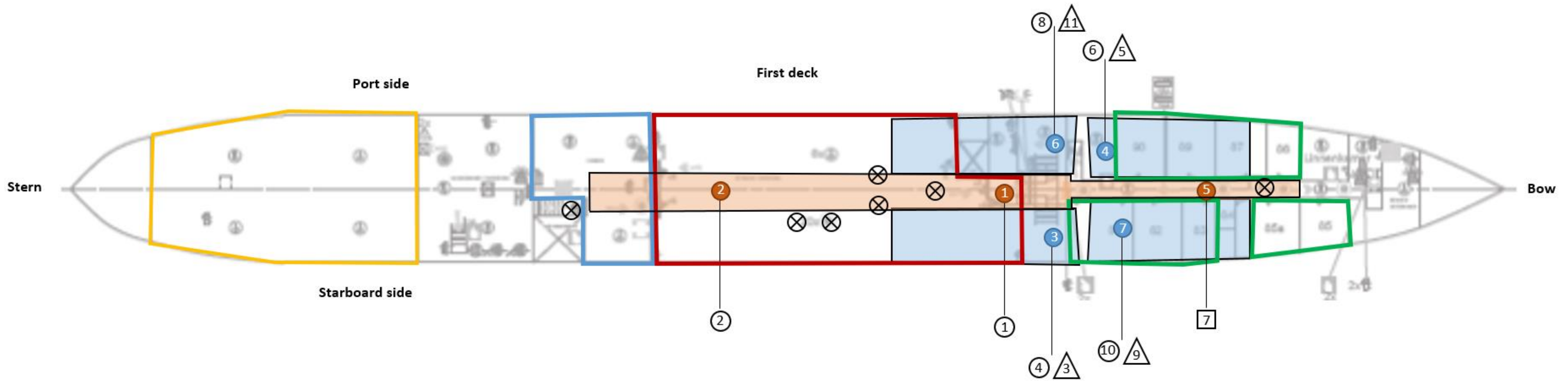

Legend

- Engine room
- Galley
- Dining room
- Crew quarters
- Hatch, opened for inspection
- Manhole to freshwater tank
- Manhole to sewage tank
- Freshwater tank
- Wastewater tank

Sample sort

- ○ Swab
- □ ½ litre container
- △ 5 litre jerrycan

Sample numbering

1	Swab	Not swabbed, but inserted in water
2	Swab	Not swabbed, but inserted in water
3	Jerrycan	Two metres, from manhole towards bow
4	Swab	Under water, from a truss (sediment/silt)
5	Jerrycan	Three metres from manhole towards bow/starboard (midships)
6	Swab	Under water, from a truss (sediment/silt)
7	Container ½ litre	From manhole
8	Swab	Pit in the wall on portside (midships) where liquid drips in the freshwater tank
9	Jerrycan	One metre from manhole towards stern
10	Swab	Hole in the wall on portside (midships) where traces of liquid dripping are visible
11	Jerrycan	Two metre from manhole towards starboard (midships)

Map 2: vertical cross-section of the ship.
Cross-sectional view of the ship, with brown colour indicating the wastewater tank and in blue the freshwater tanks.

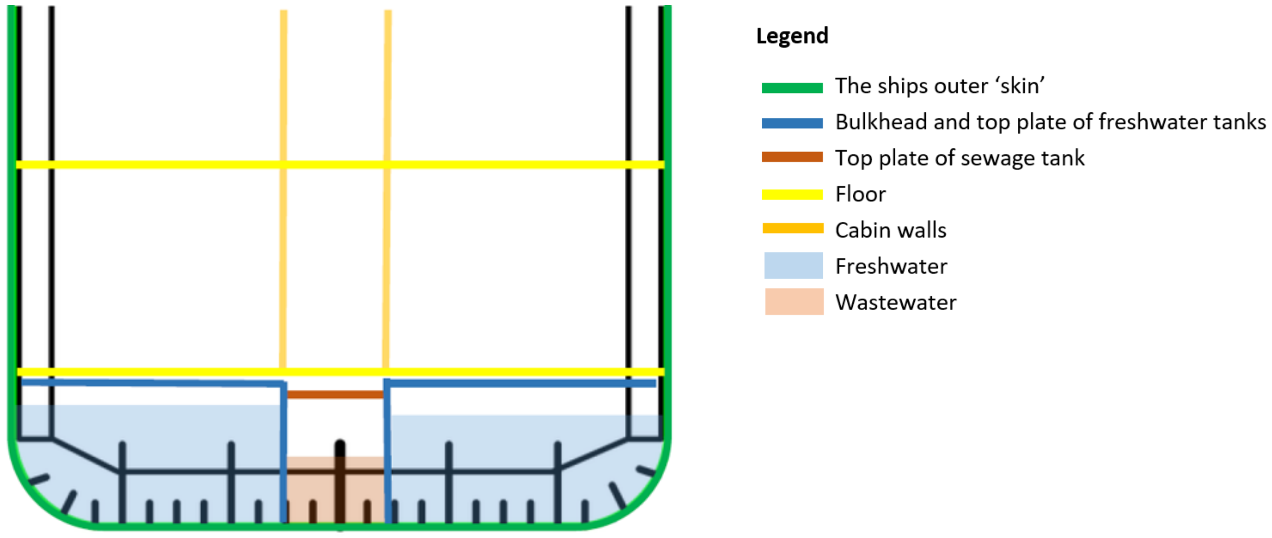

Appendix 2: pictures of ship inspection

Photographers: Jeffrey Koper, Tanja Hartog, Freek Besterman.

Freshwater tank, seen from manhole

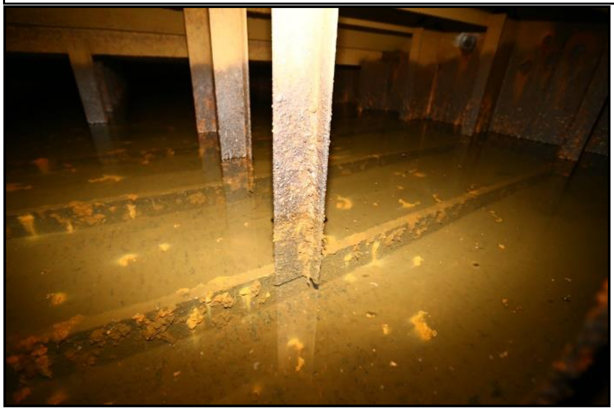

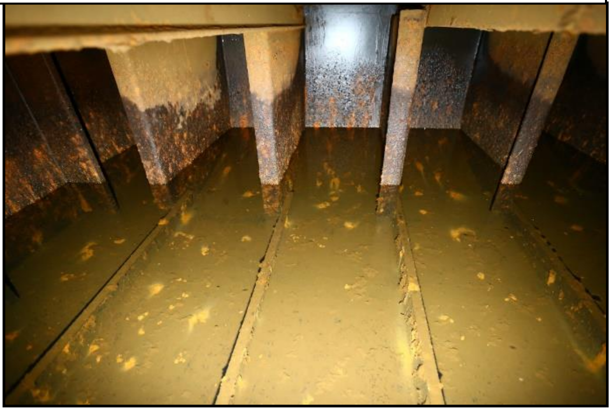

Entrance to wastewater tank, found under floor

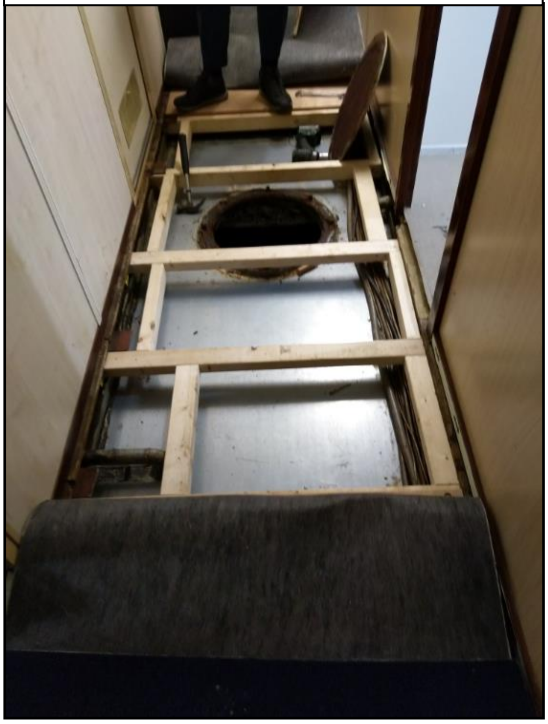

Freshwater- and wastewater tank, sharing a common wall

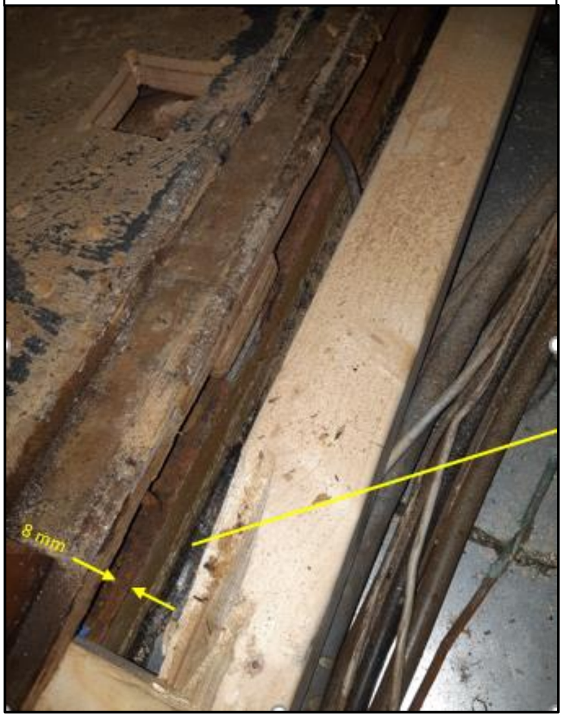

Holes in freshwater tanks, caused by corrosion

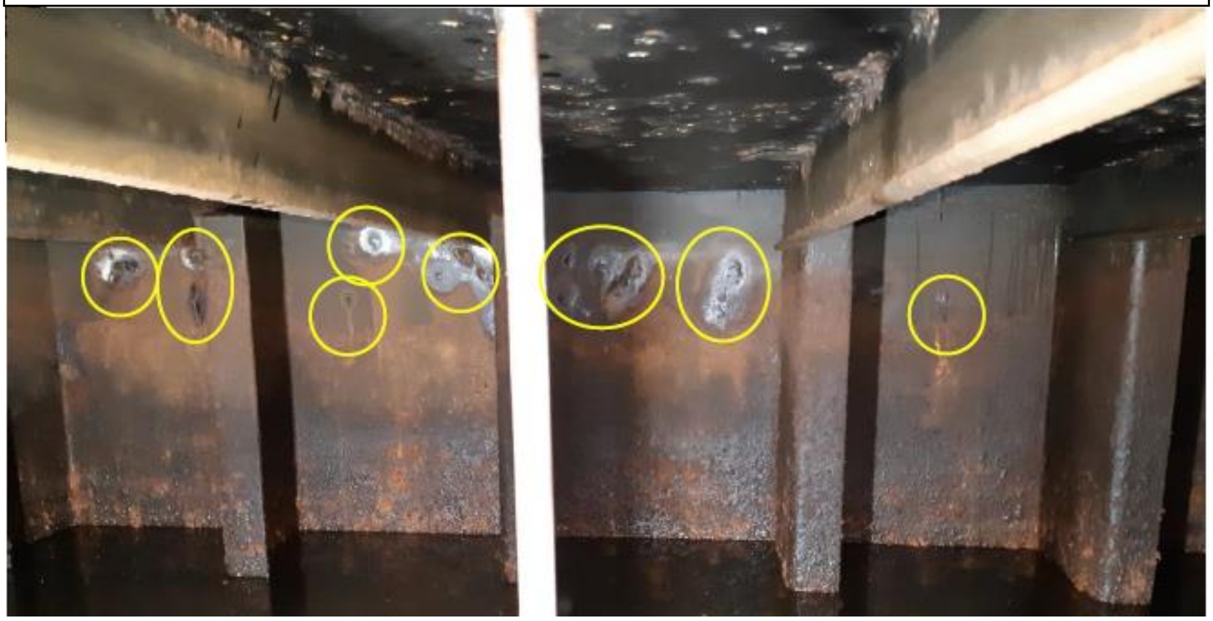

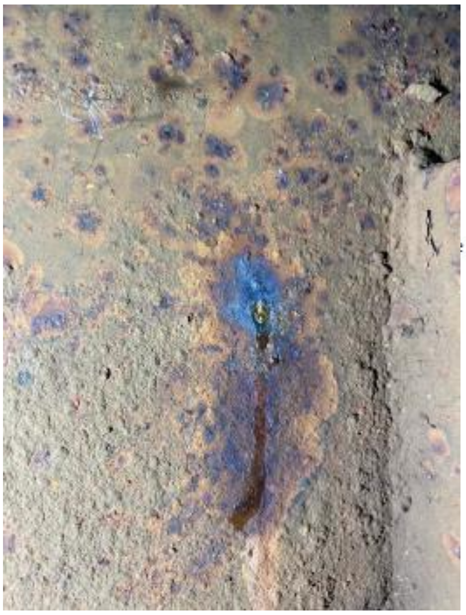

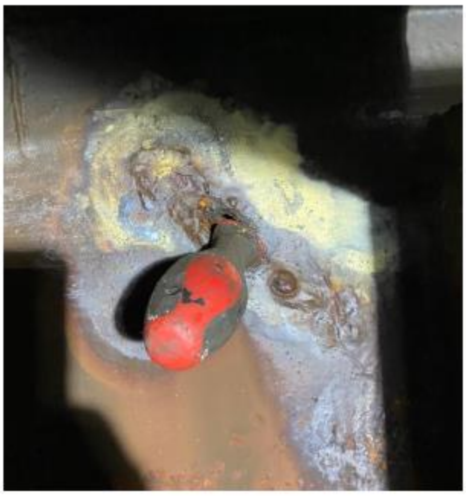

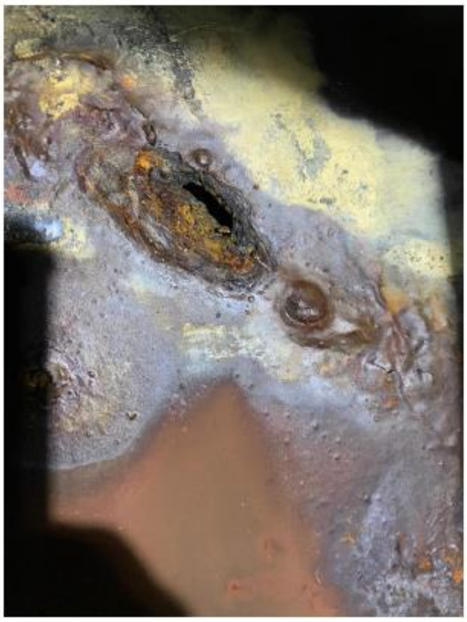

Supplement: Supplementary Material [file 23-00211_OOMS_Supplement.pdf]
